# Evaluating the Therapeutic Potential of Exercise in Hypoxia and Low-Carbohydrate, High-Fat Diet in Managing Hypertension in Elderly Type 2 Diabetes Patients: A Novel Intervention Approach

**DOI:** 10.3390/nu17030522

**Published:** 2025-01-30

**Authors:** Raquel Kindlovits, Ana Catarina Sousa, João Luís Viana, Jaime Milheiro, Bruno M. P. M. Oliveira, Franklim Marques, Alejandro Santos, Vitor Hugo Teixeira

**Affiliations:** 1 Faculty of Nutrition and Food Sciences (FCNAUP), University of Porto, 4200-465 Porto, Portugal; bmpmo@fcna.up.pt (B.M.P.M.O.); alejandrosantos@fcna.up.pt (A.S.); vhugoteixeira@fcna.up.pt (V.H.T.); 2 Research Center in Sports Sciences, Health Sciences and Human Development (CIDESD), University of Maia, 4475-690 Maia, Portugal; acsousa@umaia.pt (A.C.S.); jviana@umaia.pt (J.L.V.); 3 Exercise Medical Centre Laboratory (CMEP), 4150-044 Porto, Portugal; up198901442@edu.fade.up.pt; 4 Centre of Research, Education, Innovation and Intervention in Sport (CIFI2D), Faculty of Sport, University of Porto, 4200-540 Porto, Portugal; 5 Laboratory of Artificial Intelligence and Decision Support, Institute for Systems and Computer Engineering, Technology and Science (LIAAD, INESC-TEC), 4200-465 Porto, Portugal; 6 Laboratory of Biochemistry, Department of Biological Sciences, UCIBIO, REQUIMTE, Faculty of Pharmacy, University of Porto, 4050-313 Porto, Portugal; franklim@ff.up.pt; 7 Institute for Research and Innovation in Health (i3S), 4200-135 Porto, Portugal; 8 Research Center in Physical Activity, Health and Leisure (CIAFEL), Faculty of Sports (FADEUP), University of Porto, 4200-540 Porto, Portugal; 9 Laboratory for Integrative and Translational Research in Population Health (ITR), 4050-600 Porto, Portugal

**Keywords:** hemoglobin, blood pressure, normobaric hypoxia, carbohydrates, exercise, diabetes, elderly

## Abstract

Background/Objectives: Type 2 diabetes mellitus (T2DM) is a chronic condition marked by hyperglycemia, which can affect metabolic, vascular, and hematological parameters. A low-carbohydrate, high-fat (LCHF) diet has been shown to improve glycemic control and blood pressure regulation. Exercise in hypoxia (EH) enhances insulin sensitivity, erythropoiesis, and angiogenesis. The combination of LCHF and EH may offer a promising strategy for managing T2DM and hypertension (HTN), although evidence remains limited. This study aimed to assess the effects of an eight-week normobaric EH intervention at 3000 m simulated altitude combined with an LCHF diet on hematological and lipid profiles, inflammation, and blood pressure in older patients with T2DM and HTN. Methods: Forty-two diabetic patients with HTN were randomly assigned to three groups: (1) control group (control diet + exercise in normoxia), (2) EH group (control diet + EH), and (3) intervention group (EH+LCHF) Baseline and eight-week measurements included systolic, diastolic, and mean blood pressure (SBP, DBP, MAP), hematological and lipid profiles, and inflammation biomarkers. Results: Blood pressure decreased after the intervention (*p* < 0.001), with no significant differences between groups (SBP: *p* = 0.151; DBP: *p* = 0.124; MAP: *p* = 0.18). No differences were observed in lipid profile or C-reactive protein levels (*p* > 0.05). Mean corpuscular hemoglobin (MCH) increased in the EH group (*p* = 0.027), while it decreased in the EH+LCHF group (*p* = 0.046). Conclusions: Adding hypoxia or restricting carbohydrates did not provide additional benefits on blood pressure in T2DM patients with HTN. Further elucidation of the mechanisms underlying hematological adaptations is imperative. Trial registration number: NCT05094505.

## 1. Introduction

The International Diabetes Federation estimated that the prevalence of diabetes was 10.5% in 2021 and will increase to 11.3% by 2030 and 12.2% by 2040. This represents a total of 451 million adults worldwide with a diabetes diagnosis in 2017 and a projected increase to 693 million by 2045 [1]. Diabetes is a metabolic disease that shows itself clinically as chronic hyperglycemia. Poorly controlled or uncontrolled diabetes could be associated with many physiological processes that may affect lipid metabolism, regulation of inflammation, vasodilatation, vascular, immunological, and hematological parameters [2]. In light of this, hypertension (HTN), defined by systolic blood pressure ≥140 mmHg or diastolic blood pressure ≥90 mmHg [3], is prevalent in over 50% of individuals with type 2 diabetes (T2DM) and significantly escalates the risk of cardiovascular diseases (CVD) by fourfold compared to normotensive non-diabetic individuals [4]. Notably, individuals with T2DM who also have HTN at the time of diagnosis exhibit elevated rates of mortality and cardiovascular events, particularly pronounced in older populations [5], suggesting that much of this excess risk is attributable to coexistent HTN [6] and underscoring the critical need for effective therapeutic strategies [7].

Lifestyle medicine is a multi-disciplinary approach which mainly deals with prevention, treatment, and research on the main chronic pathologies linked to modifiable environmental factors, such as inadequate diet and sedentarism [8]. Most T2DM patients are not active [9], and a sedentary lifestyle, together with poor nutrition, are considered as the major risk factors for T2DM and its complications [10]. Current international guidelines recommend aerobic and resistance exercise training for T2DM patients for improving blood lipids, inflammation, and glycemic and blood pressure control [11]. The consensus of leading organizations such as the European Society of Cardiology [11], Belgian Physical Therapy Association [12], American College of Sports Medicine [13], American Diabetes Association [13], and Exercise and Sports Science Australia [14] advocates for a combined approach involving aerobic and resistance training. Supervised exercise programs are highlighted as particularly effective modalities for improving weight loss, blood pressure, and glycemia in T2DM patients [14].

Hyperglycemia in T2DM was negatively correlated with some hematological indices, such as red blood cells (RBC), hemoglobin (Hb), and mean corpuscular hemoglobin (MCH), due the augmented oxidative stress [15]. Improvements in these biomarkers, mainly by the production of erythropoietin (EPO) and its consequent erythropoiesis, can be helpful physiological mechanisms, but do not occur as a result of an exercise training in normoxia [16]. Conversely, adding hypoxia to exercise promotes relevant hematological adaptations [16,17]. Recent investigations have shown promising outcomes in elderly patients with CVD [18] and in individuals with T2DM [19] performing exercise in hypoxia (EH) at simulated altitudes, suggesting that EH may offer additional benefits compared to exercise in normoxia.

Physiological adaptations occur in hypoxic environments [20] mainly driven by hypoxia-inducible factor 1α (HIF-1α) activation [21] and increased expression of hypoxia-responsive genes [22]. Therefore, EH-induced erythropoiesis and angiogenesis, by increasing EPO and vascular endothelial growth factor (VEGF) levels, may contribute to improved blood pressure control and enhanced tissue oxygenation [23,24,25,26]. Not least, HIF-1α promotes lowering of blood glucose levels through stimulating glycolysis and asa result of the increased expression of GLUT4 glucose transporters in muscle tissue independent of muscle work [27]. Collectively, these adaptations could potentially mitigate the vascular, hematological, and metabolic complications associated with T2DM and HTN.

The optimal dietary approach remains a subject of debate among experts [28,29]. The low-carbohydrate, high-fat (LCHF) diet has emerged as a promising therapeutic option for individuals with T2DM and HTN, endorsed by the American Diabetes Association for glycemic control and weight management [30]. Notably, LCHF diets have demonstrated efficacy in reducing blood pressure [31], even surpassing the Dietary Approaches to Stop Hypertension (DASH) diet [32], the standard recommendation for blood pressure management by the American Heart Association [29].

Furthermore, LCHF diets have also been associated with hematological adaptations, including reductions in MCH and in mean corpuscular hemoglobin concentration (MCHC) [33]. Decreases in MCH and MCHC were inversely linked with insulin resistance and high blood pressure [34]. It was previously shown that hematological changes could occur as an effect of oxidative stress promoted by T2DM [15], and that these biomarkers may also serve as predictors of the disease’s evolution [35] due to their role as determinants of blood viscosity [36]. Increased blood viscosity could contribute to the development of T2DM [37] and HTN [34,38].

Thus, this study aimed to evaluate the effects of an eight-week normobaric EH intervention at 3000 m of simulated altitude combined with an LCHF diet on the hematological and lipid profiles, inflammation, and blood pressure in older patients with T2DM and coexistent HTN. We hypothesize that the combination of chronic EH and LCHF dietary modifications will lead to improvements in blood pressure, along with associated changes in hemoglobin levels, offering novel insights into therapeutic interventions for this high-risk population.

## 2. Materials and Methods

### 2.1. Ethical Considerations

The study was approved by the Institutional Review Board of the Faculty of Nutrition and Food Sciences, University of Porto, on 23 July 2021 (Approval Number 45/2021/CEFCNAUP/2021). It was conducted following the Declaration of Helsinki for studies in humans [39]. It has been registered in the Clinical Trial database (NCT05094505).

### 2.2. Study Design

**Figure 1:**
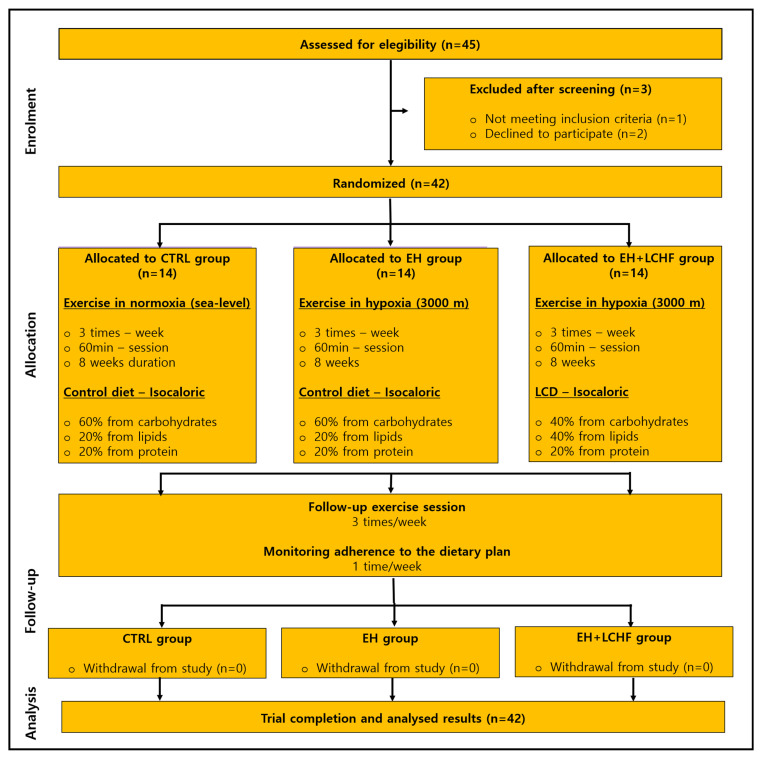
CONSORT flow diagram of the progress through the phases of the RCT. CTRL: control group: EH: exercise in hypoxia; LCHF: low-carbohydrate high-fat diet. The study had a 100% adherence rate, with no dropouts throughout the testing and intervention phases (n = 42).

This study was a controlled, single-blind, three-arm parallel randomized controlled trial (RCT). Participants were randomly allocated into three groups (*n* = 14 per group): the control group (control diet + exercise in normoxia), EH group (control diet + exercise in hypoxia), or EH+LCHF group [40]. Of the 42 participants, 24 were men and 18 were women. The experimental protocol included four phases: (1) pre-intervention assessments, (2) a familiarization period, (3) the intervention phase, and (4) post-intervention assessments [41]. Pre- and post-intervention evaluations covered hematological parameters, blood lipids, inflammation markers, and blood pressure responses. The CONSORT flow diagram is available in Figure 1.

### 2.3. Participants

Participants were eligible for inclusion if they met the following criteria: (1) aged over 65 years, of either sex, with a medical diagnosis of T2DM and HTN for at least one year; (2) hemoglobin A1c (HbA1c) levels between 6.5% and 10%; (3) systolic blood pressure ≥140 mmHg or diastolic blood pressure ≥90 mmHg; (4) stable pharmacological treatment for at least three months; (5) prior participation in supervised exercise programs within the last six months; and (6) non-smokers for at least six months. Exclusion criteria included: (a) insulin dependence; (b) uncontrolled diabetes-related microvascular or macrovascular complications, such as retinopathy, nephropathy, diabetic foot, atherosclerosis, diabetic cardiomyopathy, or recent acute myocardial infarction; (c) other uncontrolled metabolic or vascular conditions; (d) a sedentary lifestyle; or (e) physical limitations preventing exercise.

### 2.4. Dietary Plan

Each participant received a personalized dietary plan created using the Dietbox^®^ software, version 7.0. The energy content of the plan was tailored to match 100% of their estimated energy requirement (EER). The EER was determined by calculating the resting metabolic rate using the Harris–Benedict equation, recognized as the most precise method for older adults at the individual level [42], and multiplying it by the physical activity level. This level was evaluated through the International Physical Activity Questionnaire (IPAQ short form, past seven days, self-administered version for older adults), categorizing participants into three physical activity levels: low, moderate, or high [43]. Cut-point values in the “high” category considered vigorous-intensity activity on at least three days, while a moderate level of physical activity was defined by five or more days of moderate-intensity activity and/or walking for at least 30 min per day. Participants with no reported activity or insufficient activity to meet the criteria for ‘moderate’ or ‘high’ categories were classified as ‘low’ [43].

The macronutrient distribution of the control diet consisted of 60% of energy from carbohydrates, 20% from protein, and 20% from fat, while the LCHF diet provided 40% of energy from carbohydrates, 20% from protein, and 40% from fat. Both diets prioritized low-glycemic-index foods, aligning with conventional dietary guidelines [44]. Based on the classification of diets as (1) very-low-carbohydrate: less than 26% of energy intake; (2) low-carbohydrate: 26–45% of energy intake; and (3) high-carbohydrate: ≥45% of energy intake [45], we compared a low-carbohydrate diet (EH+LCHF group) with a high-carbohydrate diet (EH group). Dietary adherence was monitored through weekly 24 h recalls. Additionally, participants attended two individual appointments with a nutritionist during the eight-week intervention to promote adherence to the dietary plan.

### 2.5. Exercise Protocol

Exercise sessions, conducted either in normoxia or hypoxia (simulating an altitude of 3000 m through nitrogen dilution), were held three times per week over an eight-week intervention period in a hypoxic chamber at CMEP—Exercise Medical Center. The chamber allowed for precise control of O_2_ levels (11% to 20.97%), temperature (up to 50 °C), relative humidity (up to 80%), and simulated altitude (up to 8000 m). Altitude classification was based on the following ranges: high altitude (1500–3500 m), very high altitude (3500–5500 m), and extreme altitude (above 5500 m) [46].

Before the intervention began, participants completed six familiarization sessions over two weeks to practice exercise techniques and acclimate to the simulated conditions. The altitude was progressively increased by 500 m per session until reaching 3000 m. Exercise intensity was standardized at 75% of the heart rate reserve, as determined by a pre-intervention cardiopulmonary exercise test (CPET). Heart rate and oxygen saturation were continuously monitored using a finger pulse oximeter (Globus YM201, Milan, Italy), and the Borg Rating of Perceived Exertion (RPE) was recorded after each session [47]. All sessions occurred at a consistent time of day (±1 h), with at least 48 h of recovery between visits.

Each session lasted approximately 60 min, starting with a 5 min warm-up consisting of body mobilization and dynamic stretching. This was followed by 40 min of moderate-intensity aerobic exercise, alternating every 9 min between a cycle ergometer and a treadmill (both from Life Fitness, Chicago, IL, USA), with a 1 min rest between transitions. Strength exercises targeting different muscle groups (pectoral, shoulders, back, arms, thighs, legs, and abdominals) were performed at the end of each session. These included three sets of 12–15 repetitions per exercise, with 1 min rest periods between sets, for a total of approximately 15 min. Strength exercises alternated weekly.

### 2.6. Measurements

In all groups, hematological parameters, blood lipids, inflammation markers, and blood pressure were evaluated at baseline and 48 h after the last exercise session (eighth week), after fasting for 12 h and without any strenuous exercise in the last 24 h and no alcohol consumption in the previous 72 h.

### 2.7. Blood Samples Analyses

Hematological markers were determined using impedance and fluorescence flow cytometry, including erythrocytes (L), hemoglobin (g/dL), hematocrit (%), mean corpuscular hemoglobin (MCH, pg), mean corpuscular hemoglobin concentration (MCHC, g/dL), red cell distribution width (RDW, %), leukocytes (L), neutrophils (%), eosinophils (%), basophils (%), lymphocytes (%), monocytes (%), and platelets (L). Lipid parameters, using the spectrophotometry enzymatic method, included total cholesterol (mmol/L), high-density lipoprotein cholesterol (HDL-c, mmol/L), low-density lipoprotein cholesterol (LDL-c, mmol/L), triglycerides (TAG, mmol/L), and the CRP (mg/dL) inflammatory marker, which were collected and analyzed using the latex-enhanced immunoturbidimetry method.

### 2.8. Blood Pressure

Systolic, diastolic, and mean blood pressure (SBP, DBP, and MAP) were measured using an automated sphygmomanometer (Dinamap Pro, Little Chalfont, Buckinghamshire, UK) in the left arm, with the participant in a sitting position, and after ten minutes of rest on the same day as blood sample collection. The measurements were performed in triplicate, and the average was expressed in mmHg.

### 2.9. Statistical Analysis

Sample and power calculations for this study were based on changes in hemoglobin A1c observed in the RCTs included in the meta-analysis by Zuuren et al. [48]. To detect an effect size of Cohen’s d = 1.14 with 80% power (α = 0.05, two-tailed), G*Power software, version 3.1 [49] estimated that 28 participants would be required for a paired-samples *t*-test, totaling 42 individuals across the three experimental groups. Accounting for a 20% dropout rate, 48 participants were recruited. Data were reported as mean (standard deviation, SD). Normality was evaluated using the Shapiro–Wilk test. For continuous variables that did not follow a normal distribution, logarithmic transformations were applied. When skewness was positive, the transformation y = log(x − L) was used, where L < minimum of x; for negative skewness, y = log (H − x), where H > maximum of x. Diastolic blood pressure, however, was transformed using the Box–Cox method with a parameter λ = 0.75. To analyze changes in hematological markers, CRP, lipid profile, and blood pressure over the eight-week intervention (baseline vs. eighth week) and to assess differences in adaptations over time and among groups, a two-way repeated-measures ANOVA was performed. When significant main or interaction effects were observed, Tukey’s post hoc test was applied for multiple pairwise comparisons. All statistical analyses were conducted using SPSS Statistics software, version 28.0 (IBM, Chicago, IL, USA), and the results were considered significant at *p* ≤ 0.05.

## 3. Results

### 3.1. Baseline Characteristics

The baseline characteristics of the participants are shown in Table 1. None of the participants were injured or had adverse responses to the EH or LCHF diet. The groups did not differ significantly at this moment, either in terms of gender or age.

### 3.2. Dietary and Exercise Interventions

Detailed information about the exercise and diet interventions has been previously published [41]. In summary, the EH+LCHF group consumed significantly fewer carbohydrates (*p* < 0.001) and more total fat (*p* < 0.001), with no notable differences between groups in energy intake (*p* = 0.69) or fiber consumption (*p* = 0.49). Over the eight weeks of exercise sessions, the groups exercising in hypoxia (EH and EH+LCHF) showed significantly lower average oxygen saturation compared to the CTRL group (*p* < 0.001), while heart rate values were comparable across all groups (*p* = 0.63). Additionally, perceived exertion (RPE) was recorded, with the EH and EH+LCHF groups reporting the highest levels of effort (*p* < 0.001), reflecting the subjective intensity of the physical exercise.

### 3.3. Cardiovascular Risk Factors

Cardiovascular risk factors were evaluated by lipid profile and CRP. No effects from baseline to post-intervention were observed on total cholesterol (*p* = 0.08), HDL-c (*p* = 0.987), LDL-c (*p* = 0.501) or TAG (*p* = 0.435), nor among the three groups (*p* = 0.135; p00.511; *p* = 0.119; *p* = 0.518), respectively. CRP did not show differences from baseline to the eighth week of intervention (*p* = 0.090), and did not differ among groups (*p* = 0.66, Table 2).

### 3.4. Hematological Parameters

The hemogram of the study participants is shown in Table 3. There were no significative differences from pre- to post-interventions regarding erythrocytes (*p* = 0.585), Hb (*p* = 0.355), hematocrit (*p* = 0.460), RDW (*p* = 0.059), leukocytes (*p* = 0.999), neutrophils (*p* = 0.192), eosinophils (*p* = 0.863), basophils (*p* = 0.691), lymphocytes (*p* = 0.279%), monocytes (*p* = 0.303), or platelets (*p* = 0.105). There were no differences between the moments for MCH (*p* = 0.733) and MCHC (*p* = 0.669), but MCH increased only in the EH group (*p* = 0.027), and MCHC was reduced only in the EH+LCHF group (*p* = 0.046).

### 3.5. Blood Pressure

SBP, DBP, and MAP were significantly reduced (*p* < 0.001) from pre- to post-intervention (Table 4). While SBP, DBP, and MAP values decreased after interventions, post hoc analysis revealed that these changes did not reach statistical significance between groups (*p* = 0.151; *p* = 0.124; *p* = 0.18, respectively). No differences were found in resting heart rate regarding moments (*p* = 0.090) or groups (*p* = 0.660).

## 4. Discussion

This is the first RCT examining the responses to chronic EH with and without an LCHF diet on hematological and lipid profile, inflammation, and blood pressure in patients with T2DM and coexistent HTN. The study’s main findings revealed an increase in MCH in patients who exercised in hypoxia, while a reduction in MCH concentration was observed in patients following an LCHF diet. Additionally, the study identified a significant reduction in HTN following eight weeks of exercise in normoxia and hypoxia, with no substantial disparity in the efficacy of EH compared to normoxia, irrespective of dietary carbohydrate content, which contradicts our hypothesis. These findings align with prior research indicating comparable blood pressure outcomes between EH and normoxia-based exercise interventions in older individuals over an eight-week period with the same oxygen levels (~15% of FIO_2_) [50].

However, contrasting results from existing studies suggest superior effects of regular EH on blood pressure regulation. For instance, moderate exercise at a natural altitude of 1700 m exhibited notable reductions in SBP and DBP in individuals with metabolic syndrome over a three-week period [26]. Reductions of 10 mmHg and 7 mmHg in SBP were observed after four weeks of chronic EH at 16.4 and 14.5% of FIO_2_, respectively [23,24]. In contrast, other researchers observed a reduction only in DBP after 13 weeks of aerobic and strength exercise in normobaric hypoxia simulating 2000–3350 m altitude [25]. It is noteworthy that most of these studies reported an improved body composition after EH intervention [23,25,26], a crucial factor in decreasing blood pressure via exercise [51].

Moreover, there is evidence suggesting that older individuals may exhibit resistance to the reduction in exercise-induced blood pressure [51], and this resistance appears to be closely tied to changes in body composition [23,51]. Published data from the current RCT showed reductions in weight, body mass index (BMI), and body fat after eight weeks of interventions [41], and there were no significant additional benefits observed in the groups exercising in hypoxia compared to those exercising in normoxia. Similarly, our investigation into blood pressure failed to reveal any notable advantages of exercising in hypoxia over normoxia, irrespective of dietary carbohydrate content. In agreement, it was demonstrated that an eight-month training program led to improvements in weight, BMI, and waist and hip circumference over time, with no discernible differences between the normoxia and hypoxia exercise groups [52].

Despite observing similar improvements in blood pressure with both normoxic and hypoxic exercise interventions, along with comparable levels of Hb mass, MCH, and MCHC before and after chronic EH and LCHF diet interventions, our study revealed higher MCH levels in the EH group. It is widely acknowledged that achieving a substantial increase in hemoglobin content requires an adequate hypoxic dose of >12 h per day at a sufficient altitude for >21 days (approximately 300 h) [53,54]. This suggests that the similarity in hemogram biomarkers from pre- to post-eight weeks of intervention in the current study may be attributed to the short exposure to simulated altitude, specifically 3 h per week and 24 h during the total intervention period.

Compensatory elevation in MCH within a specific range is recognized as a fundamental physiological response to high-altitude hypoxia [55], enhancing blood’s oxygen-carrying capacity and improving tissue oxygenation without increasing cardiac output [56]. This adjustment translates to an increase of approximately 0.30–0.47 g/dL per 1000 m of altitude [50]. However, when Hb production significantly exceeds the reference range, an increase in cardiac output is required to sustain oxygen transport [56], potentially contributing to blood pressure elevation. Given that Hb production remained within the normal range proposed for sea level [57] and when corrected by high altitudes [58], it appears that the increase in MCH production within the minimum cut-off values in patients who exercised in hypoxia was not sufficient to induce significant changes in blood pressure compared to exercise in normoxia, at least with 24 h of hypoxic exposure at a 3000 m simulated altitude.

Contrary to our findings, previous studies have demonstrated improvements in SPB and DBP following long-term exposure (approximately 6 and 12 months) to natural high altitudes, accompanied by increased Hb levels [59]. Other authors have found similar improvements after 15 sessions of hypoxia exposure (14–10% FiO_2_) compared to normoxia [60]. This discrepancy may be attributed to altered hydration statuses in the EH group, with increases in Hb content when dehydrated [57]. However, the mean globular volume values in the current study were within the normal reference range in all participants and, therefore, do not support this hypothesis.

Additionally, existing evidence has demonstrated that incorporating hypoxia into exercise, even during short periods and at moderate high altitudes, yields greater benefits in blood pressure, independent of hematological parameters. This suggests the involvement of alternative mechanisms beyond MCH in this response [23,24,25]. Chronic EH has been linked to reduced arterial stiffness [61] and improvements in metabolic risk factors such as body fat and insulin resistance [62], all of which play a role in regulating blood pressure. EH also induces vasodilation and lowers blood pressure in patients with T2DM [63]. These effects are, in part, mediated by increased HIF-1α protein expression, which is inversely associated with systemic blood pressure [20]. HIF-1α also triggers VEGF activation [64], impacting blood pressure regulation via nitric oxide synthase expression and nitric oxide activity [63]. Unfortunately, we did not evaluate HIF-1α or VEGF levels in our study.

It is well known that elevated levels of Hb, MCH, and MCHC are associated with increased blood viscosity [65], which induces decreased blood flow to skeletal muscles and fat tissues, contributing to peripheral vascular resistance and elevating the risk of T2DM development, interfering with insulin-mediated glucose uptake [37], and potentially elevating blood pressure [66], thus contributing to the development of HTN [34,38]. While the EH group exhibited increased MCH levels within normal ranges, restricting carbohydrate intake appeared to decrease MCH concentration, albeit within appropriate levels [57,58]. Consistent with this, prior research reported that a LCHF diet over twelve weeks reduced iron intake and lowered both MCH and MCHC in male endurance athletes [33]. This reduction could be attributed to increased inflammation associated with the LCHF diet, affecting the iron regulatory hormone hepcidin [67]. Hepcidin production is stimulated by iron and inflammation, but inhibited by hypoxia [68]. Considering that Hb comprises nearly 70% of iron in the body, the LCHF diet’s reduction in iron consumption, coupled with the hypoxia-induced decrease in hepcidin production, could explain the reduced MCHC values observed in our study.

Moreover, MCHC has been linked to metabolic disorders, with levels increased in obesity and decreased by medications improving cellular insulin sensitivity [68]. Prediabetic patients with higher Hb content have been shown to exhibit impaired blood pressure, HDL-c levels, and waist circumference [69], contributing to a proinflammatory state and worsening metabolic dysfunction and CVD development [70]. Although our study found no differences in lipid profile or CRP levels among times and groups, evidence suggests a positive association between reduced MCHC and carbohydrate-restricted diets, with consequent improvements in insulin resistance and blood pressure, highlighting a crucial mechanism for further exploration in patients with T2DM and coexistent HTN.

In summary, while EH interventions did not demonstrate superior benefits in reducing blood pressure compared to normoxia-based exercise, our findings underscore the intricate interplay between exercise, diet, and hematological parameters in managing metabolic disorders and hypertension in patients with T2DM. Further research is needed to elucidate the underlying mechanisms and optimize therapeutic strategies for this population.

### 4.1. Strengths and Limitations

The study had a 100% adherence rate, with no dropouts throughout the testing and intervention phases, surpassing the general cutoff point for sufficient adherence in older adults by 30 percentage points (pp) [71]. To uphold participation levels, the research team maintained constant contact with participants throughout the eight-week intervention period, motivating them to complete their participation regularly during the eight weeks of intervention. This proactive approach helped motivate participants, ensuring their consistent involvement in the study. Recognizing potential barriers to attendance, such as transportation challenges, the study provided chauffeured transportation for participants to attend exercise sessions at the clinic, removing logistical hurdles. Regular meetings were convened to assess food consumption and address any queries, fostering correct adherence to the prescribed dietary plan among all participants. This personalized support contributed to the overall adherence success of the intervention.

Some limitations should also be mentioned. Due to logistical and budgetary constraints, the study was limited to an eight-week duration, resulting in a total of 24 h of hypoxia exposure. While sufficient for certain outcomes, this timeframe may have been insufficient to induce substantial changes in hematological parameters [54], such as Hb, MCH and MCHC, and their influence on blood pressure regulation. The reasons mentioned above precluded the determination of key biomarkers, including HIF-1α, VEGF, iron deficiency markers, and hepcidin levels. These markers are vital for understanding the mechanistic underpinnings of the intervention and its effects on blood pressure regulation.

### 4.2. Future Research

Future research should delve deeper into key biomarkers, including HIF-1α, VEGF, EPO, iron deficiency markers, and hepcidin levels. These biomarkers, along with a prolonged duration of hypoxia exposure, will reveal their role in blood pressure regulation in type 2 diabetic patients undergoing EH and LCHF dietary interventions. Additionally, it is expected that, with an increase close to 300 h of exposure to hypoxia, substantial changes in the hematological profile and blood pressure will be achieved in patients with T2DM.

## 5. Conclusions

In conclusion, diets and exercise lowered HTN, with no additional benefits from added hypoxia or restricted carbohydrates. Future research is needed to provide a deeper understanding of the precise mechanisms underlying hematological adaptations and their subsequent impact on blood pressure regulation. Additional elucidation of these mechanisms is imperative for a comprehensive understanding of the therapeutic potential and optimization of EH and LCHF dietary interventions in managing related HTN complications in individuals with T2DM.

## Figures and Tables

**Table 1:** Baseline characteristics of the participants.

Variables	CTRL Group	EH Group	EH+LCHF Group	*p*-Value
Gender (male:female)	7:7	8:6	9:5	0.747
Age (years)	74.4 (3.6)	71.6 (3.8)	70.7 (4.0)	0.110
Body mass index (kg/m^2^)	29.4 (4.1)	28.3 (4.0)	29.3 (3.4)	0.707
Hemoglobin A1c (%)	6.9 (0.8)	7.1 (0.7)	6.8 (0.5)	0.647
Fasting glucose (mg/dL)	118.7 (27.8)	117.9 (22.3)	108.2 (19.7)	0.435
Systolic blood pressure (mmHg)	154.7 (20.9)	142.3 (18.2)	148.0 (18.9)	0.254
Diastolic blood pressure (mmHg)	77.5 (8.1)	76.3 (11.7)	82.9 (16.3)	0.347
Mean arterial blood pressure (mmHg)	107.9 (16.9)	98.7 (13.6)	107.1 (15.8)	0.234

A total of 42 subjects participated in this study. All values are presented as the mean (SD). Abbre-viations: CTRL group: control group; EH group: exercise in hypoxia group; EH+LCHF group: ex-ercise in hypoxia + low-carbohydrate, high-fat diet group.

**Table 2:** Lipid profile and inflammation marker pre- and post-eight weeks of interventions.

Variables	CTRL Group			EH Group			EH+LCHF Group			*p*-Value	
	Pre	Post	Δ	Pre	Post	Δ	Pre	Post	Δ	Moments	Groups
Cholesterol (mmol/L)	181.2 (49.5)	188.1 (51.8)	2.4 (17.7)	178.9 (30.1)	161.9 (23.2)	7.1 (6.7)	158.5 (46.4)	143.2 (42.7)	7.3 (16.4)	0.082	0.135
HDL-c (mmol/L)	53.8 (11.7)	52.5 (10.6)	0.5 (2.2)	52.3 (13.1)	52.0 (9.4)	0.1 (2.8)	56.2 (14.0)	57.2 (13.9)	0.4 (1.4)	0.987	0.511
LDL-c (mmol/L)	100.4 (43.2)	112.5 (48.4)	3.6 (14.3)	104.5 (29.2)	93.8 (19.1)	3.3 (6.4)	87.7 (36.6)	77.7 (28.4)	3.5 (8.3)	0.501	0.119
TAG (mmol/L)	134.6 (61.5)	133.8 (46.4)	1.2 (9.4)	136.5 (59.6)	126.8 (47.1)	2.1 (9.0)	96.2 (29.8)	91.2 (29.6)	2.5 (9.9)	0.435	0.518
CRP (mg/dL)	1.8 (2.4)	3.3 (6.4)	0.1 (0.3)	1.9 (2.1)	1.6 (1.7)	0.1 (0.1)	3.4 (4.7)	1.7 (1.3)	0.2 (0.3)	0.155	0.19

Abbreviations: CTRL group: control group; EH group: exercise in hypoxia group; EH+LCHF group: exercise in hypoxia + low-carbohydrate high-fat diet group; HDL-c: high-density lipoprotein cholesterol; LDL-c: low-density lipoprotein cholesterol; TAG: triglycerides; CRP: C-reactive protein. ∆ = Changes from baseline to the eighth week.

**Table 3:** Hemogram with platelets pre- and post-eight weeks of interventions.

Variables	CTRL Group			EH Group			EH+LCHF Group			*p*-Value	
	Pre	Post	Δ	Pre	Post	Δ	Pre	Post	Δ	Moments	Groups
Erythrocytes (L)	4.7 (0.4)	4.7 (0.5)	0.1 (0.1)	4.5 (0.4)	4.5 (0.5)	0.1 (0.2)	4.6 (0.51)	4.7 (0.5)	0.1 (0.2)	0.585	0.122
Hemoglobin (g/dL)	14.2 (1.0)	14.2 (1.2)	0.1 (0.5)	13.9 (1.3)	13.7 (1.4)	0.1 (0.6)	13.8 (1.6)	13.7 (1.7)	0.1 (0.5)	0.355	0.668
Hematocrit (%)	42.7 (3.1)	42.7 (3.5)	0.1 (1.3)	41.7 (3.6)	41.1 (4.3)	0.5 (1.9)	41.9 (4.1)	41.9 (5.0)	0.1 (1.6)	0.460	0.611
MCH (pg)	29.9 (2.2)	29.2 (2.2)	0.7 (2.7)	30.2 (1.2)	31.3 (1.7)	1.1 (1.3)	29.7 (1.8)	29.6 (1.8)	0.1 (0.3)	0.733	0.027 #
MCHC (g/dL)	33.3 (1.3)	33.4 (1.3)	0.1 (0.3)	33.3 (1.1)	33.4 (1.3)	0.1 (0.3)	32.9 (0.9)	32.6 (0.8)	0.2 (0.5)	0.669	0.046 #
RDW (%)	12.5 (0.5)	12.6 (0.7)	0.1 (0.3)	12.8 (0.5)	12.8 (0.5)	0.1 (0.1)	13.4 (0.6)	13.5 (0.6)	0.1 (0.4)	0.059	0.911
Leukocytes (L)	7.4 (2.1)	7.1 (1.9)	0.2 (1.0)	6.1 (1.3)	6.3 (1.1)	0.2 (1.0)	6.1 (1.4)	5.8 (1.1)	0.3 (0.9)	0.999	0.156
Neutrophils (%)	62.2 (8.0)	60.3 (10.1)	1.8 (5.2)	63.1 (7.2)	63.9 (7.7)	0.9 (5.4)	61.5 (7.5)	59.3 (7.4)	2.1 (4.6)	0.192	0.241
Eosinophils (%)	2.6 (1.4)	2.5 (1.4)	0.1 (0.4)	2.2 (1.1)	1.9 (1.1)	0.2 (0.6)	2.4 (1.4)	2.8 (1.1)	0.37 (1.1)	0.863	0.118
Basophils (%)	0.5 (0.1)	0.4 (0.1)	0.1 (0.1)	0.6 (0.2)	0.7 (0.2)	0.1 (0.2)	0.6 (0.2)	0.6 (0.3)	0.1 (0.2)	0.691	0.138
Lymphocytes (%)	28.6 (6.3)	30.4 (9.1)	1.7 (4.7)	28.1 (6.9)	27.1 (7.1)	0.9 (5.1)	28.7 (7.2)	30.3 (8.1)	1.6 (4.4)	0.279	0.260
Monocytes (%)	5.9 (1.4)	6.2 (1.6)	0.2 (1.3)	5.9 (1.1)	6.1 (1.1)	0.2 (0.6)	6.7 (1.2)	6.90 (1.6)	0.1 (1.1)	0.303	0.967
Platelets (L)	218.2 (38.0)	212.8 (35.4)	5.4 (13.8)	213.5 (43.3)	227.4 (31.2)	13.8 (27.1)	219.1 (35.2)	223.2 (35.9)	4.1 (32.0)	0.105	0.066

# = MCH (pg): EH group increased from CTRL and EH+LCHF groups; MCHC (g/dL): EH+LCHF group decreased from EH and CTRL groups. Abreviations: CTRL group: control group; EH group: exercise in hypoxia group; EH+LCHF group: exercise in hypoxia + low-carbohydrate, high-fat diet group; MCH: mean corpuscular hemoglobin; MCHC: mean corpuscular hemoglobin concentration, RDW: red cell distribution width. ∆ = Changes from baseline to the eighth week.

**Table 4:** Blood pressure measures pre- and post-eight weeks of interventions.

Variables	CTRL Group			EH Group			EH+LCHF Group			*p*-Value	
	Pre	Post	Δ	Pre	Post	Δ	Pre	Post	Δ	Moments	Groups
SBP (mmHg)	154.7 (20.9)	142.5 (16.6)	12.2 (10.2)	142.3 (18.2)	124.5 (14.6)	17.7 (15.6)	148.0 (18.9)	126.5 (18.6)	21.5 (10.6)	<0.001 *	0.151
DBP (mmHg)	77.5 (8.1)	72.2 (7.7)	5.4 (5.2)	76.3 (11.7)	71.8 (12.0)	4.1 (6.6)	82.9 (16.3)	71.4 (8.4)	9.0 (8.9)	<0.001 *	0.124
MAP (mmHg)	107.9 (16.9)	102.9 (10.2)	5.0 (13.3)	98.7 (13.6)	87.0 (17.8)	11.7 (15.6)	107.1 (15.8)	92.0 (12.1)	15.1 (12.3)	<0.001 *	0.158
RHR (bpm)	68.5 (9.5)	69.0 (9.7)	0.5 (5.2)	64.7 (10.5)	67.3 (10.1)	2.4 (8.1)	62.1 (8.1)	65.0 (9.5)	2.8 (8.3)	0.090	0.660

Abbreviations: CTRL group: control group; EH group: exercise in hypoxia group; EH+LCHF group: exercise in hypoxia + low-carbohydrate, high-fat diet group; SBP: systolic blood pressure; DBP: diastolic blood pressure; MAP: mean arterial blood pressure; RHR: resting heart rate. ∆ = Changes from baseline to the eighth week. The symbol “*” indicates significant differences between results pre- and post- eight weeks of intervention.

## Data Availability

Data supporting the findings of this study are available upon request from the corresponding author, subject to ethical considerations.
